# Physapruin A Enhances DNA Damage and Inhibits DNA Repair to Suppress Oral Cancer Cell Proliferation

**DOI:** 10.3390/ijms23168839

**Published:** 2022-08-09

**Authors:** Tzu-Jung Yu, Ching-Yu Yen, Yuan-Bin Cheng, Chia-Hung Yen, Jiiang-Huei Jeng, Jen-Yang Tang, Hsueh-Wei Chang

**Affiliations:** 1 Graduate Institute of Natural Products, Kaohsiung Medical University, Kaohsiung 80708, Taiwan; 2 Department of Oral and Maxillofacial Surgery, Chi-Mei Medical Center, Tainan 71004, Taiwan; 3 School of Dentistry, Taipei Medical University, Taipei 11031, Taiwan; 4 Department of Marine Biotechnology and Resources, National Sun Yat-sen University, Kaohsiung 80424, Taiwan; 5 School of Dentistry, College of Dental Medicine, Kaohsiung Medical University, Kaohsiung 80708, Taiwan; 6 Department of Dentistry, Kaohsiung Medical University Hospital, Kaohsiung 80708, Taiwan; 7 Department of Dentistry, National Taiwan University Hospital, Taipei 100225, Taiwan; 8 School of Post-Baccalaureate Medicine, Kaohsiung Medical University, Kaohsiung 80708, Taiwan; 9 Department of Radiation Oncology, Kaohsiung Medical University Hospital, Kaohsiung 80708, Taiwan; 10 Department of Biomedical Science and Environmental Biology, PhD Program in Life Science, College of Life Science, Kaohsiung Medical University, Kaohsiung 80708, Taiwan; 11 Institute of Medical Science and Technology, National Sun Yat-sen University, Kaohsiung 80424, Taiwan; 12 Center for Cancer Research, Kaohsiung Medical University, Kaohsiung 80708, Taiwan

**Keywords:** withanolides, oral cancer, antiproliferation, oxidative stress

## Abstract

The selective antiproliferation to oral cancer cells of *Physalis peruviana*-derived physapruin A (PHA) is rarely reported. Either drug-induced apoptosis and DNA damage or DNA repair suppression may effectively inhibit cancer cell proliferation. This study examined the selective antiproliferation ability of PHA and explored detailed mechanisms of apoptosis, DNA damage, and repair. During an ATP assay, PHA provided high cytotoxicity to two oral cancer cell lines (CAL 27 and Ca9-22) but no cytotoxicity to two non-malignant oral cells (HGF-1 and SG). This selective antiproliferation of PHA was associated with the selective generation of reactive oxygen species (ROS) in oral cancer cells rather than in non-malignant oral cells, as detected by flow cytometry. Moreover, PHA induced other oxidative stresses in oral cancer cells, such as mitochondrial superoxide generation and mitochondrial membrane potential depletion. PHA also demonstrated selective apoptosis in oral cancer cells rather than non-malignant cells in annexin V/7-aminoactinmycin D and caspase 3/7 activity assays. In flow cytometry and immunofluorescence assays, PHA induced γH2AX expressions and increased the γH2AX foci number of DNA damages in oral cancer cells. In contrast, the mRNA expressions for DNA repair signaling, including homologous recombination (HR) and non-homologous end joining (NHEJ)-associated genes, were inhibited by PHA in oral cancer cells. Moreover, the PHA-induced changes were alleviated by the oxidative stress inhibitor *N*-acetylcysteine. Therefore, PHA generates selective antiproliferation, oxidative stress, and apoptosis associated with DNA damage induction and DNA repair suppression in oral cancer cells.

## 1. Introduction

Oral cancer is one of the top 10 common cancers worldwide. Men show a greater incidence than females, increasing yearly [1]. In Taiwan, oral cancer also shows a high incidence, which is associated with three main risk factors such as betel quid, smoking, and drinking [2]. After surgery, chemoradiotherapy for oral cancer is commonly accompanied by several side effects [3]. For example, clinical platinum-based drugs and 5-fluorouracil for oral cancer have several adverse responses [4,5]. Side effects may partly be attributed to the cytotoxicity effect on normal cells. Identifying anticancer drugs with lower cytotoxicity to normal cells can improve their therapeutic application to oral cancer.

*Physalis peruviana* L., also known as goldenberry, is an edible plant species belonging to the family Solanaceae, containing about 120 species [6], commonly applied in traditional medicines in Asia and South America [7]. Over 300 natural steroidal lactones belong to withanolides [7]. At least 40 withanolides were reported from *P. peruviana* [8]. Several withanolides were reported to cause antiproliferation to some cancer cells [9,10,11,12,13,14].

To date, the anticancer effects of the *P. peruviana*-derived withanolide physapruin A (PHA) are rarely reported, although it was identified in 1993 [15]. The anticancer effects of PHA were reported in prostate and renal cancer cells [8]. However, this study mainly isolated and characterized several compounds, but simply provided IC_50_ values without investigating anticancer mechanisms. Recently, we reported the anti-breast cancer effects of PHA treatment by monitoring the inductions for oxidative stress, apoptosis, and DNA damage [16]. However, the cytotoxic effects of PHA on normal cells were not examined, but the side effect safety of PHA still needs to be considered. The selective killing potential of PHA is not reported as of yet, and the possible anticancer effects of PHA on oral cancer cells remain unclear.

Inducing DNA damage to cause cell death is a primary antiproliferation strategy for anticancer therapy [17]. Cancer cells may initiate DNA damage response (DDR) to repair the lesions for compensation to defend against drug-induced DNA damage. Cancer cells become resistant when DNA damage is repairable [18]. In addition to inflicting DNA damage, disturbance of the DNA repair machinery also plays a vital role in anticancer therapy. When the DNA repair system can be dysregulated, the anticancer drug response to cancer cells may be improved [18]. Accordingly, the strategy to impair DNA repair provides a promising approach in anticancer therapy [19,20,21,22]. Although the DNA damage effects of PHA were demonstrated in breast cancer cells [16], its impact on the DNA repair machinery is not addressed as yet.

The present study evaluated the selective killing and apoptosis effects of PHA in oral cancer cells compared to non-malignant oral cells. Moreover, the involvement of double-strand break repairs such as homologous recombination (HR) and non-homologous end joining (NHEJ) in oral cancer cells was explored.

## 2. Results

### 2.1. PHA Selectively Induces Oxidative Stress- and Apoptosis-Dependent Antiproliferation to Oral Cancer

**Figure 1:**
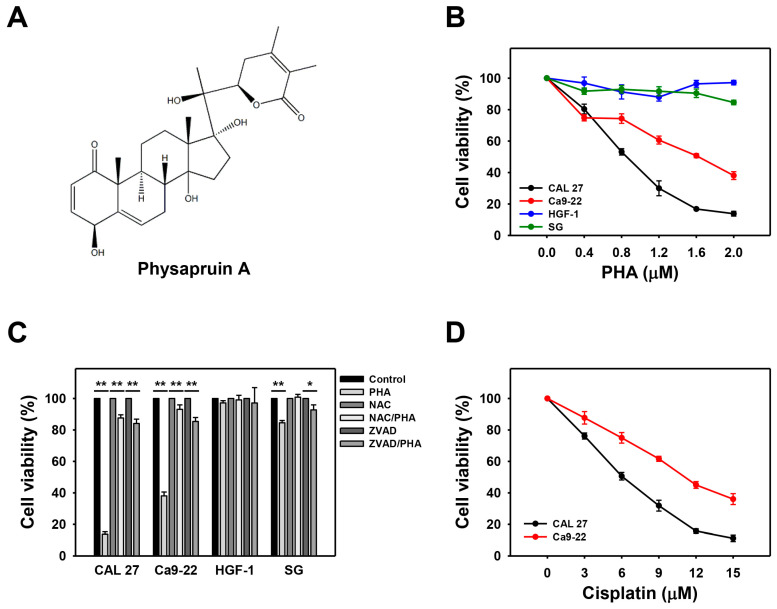
PHA selectively decreases the cell viabilities of oral cancer cells but not for non-malignant oral cells. ATP content after 24 h drug treatment was used for cell viability determination. (**A**) Structure of PHA. (**B**) Cell viability ATP assay. Two oral cancer (CAL 27 and Ca9-22) and two non-malignant oral (HGF-1 and SG) cell lines were examined. Cells were exposed to 0 (0.1% DMSO medium as control), 0.4, 0.8, 1.2, 1.6, and 2 μM PHA for 24 h. (**C**) Suppression of PHA-induced antiproliferation by *N*-acetylcysteine (NAC) or Z-VAD-FMK (ZVAD). To evaluate the recovery effect of NAC and ZVAD, NAC (10 mM for 1 h) or ZVAD (100 μM for 2 h) were added to cells before a 24 h treatment with PHA (control (0.1% DMSO medium) and 2 μM), i.e., NAC/PHA or ZVAD/PHA. (**D**) Cisplatin sensitivity to oral cancer cell lines at 24 h ATP assay. Data, means ± SDs (*n* = 3 independent experiments); * and ** indicate *p* < 0.05 and 0.001.

Based on 24 h ATP content, PHA (Figure 1A) dose-responsively inhibited the cell viability of oral cancer (CAL 27 and Ca9-22) cells, but it remained non-cytotoxic to non-malignant oral (HGF-1 and SG) cells (Figure 1B). These results suggested that PHA selectively killed oral cancer cells rather than non-malignant oral cells.

In addition, pretreatments with oxidative stress and apoptosis inhibitors (NAC and ZVAD) recovered the PHA-promoted antiproliferation of oral cancer cells (Figure 1C). These results suggested that oxidative stress and apoptosis are central factors for the selective antiproliferation of PHA acting upon oral cancer cells. For comparison, oral cancer cells showed a low sensitivity to cisplatin (Figure 1D) when compared to PHA (Figure 1B), i.e., the IC_50_s were 0.86 and 1.61 μM (PHA) and 6.02 and 11.08 μM (cisplatin) in CAL 27 and Ca9-22 cells, respectively. The PHA concentrations (0.8, 1.2, and 2 μM) at ~50, 30, and 10% viabilities for CAL 27 cells were chosen for the subsequent experiments.

### 2.2. PHA Causes Phase Changes in Cell Cycle of Oral Cancer Cells

**Figure 2:**
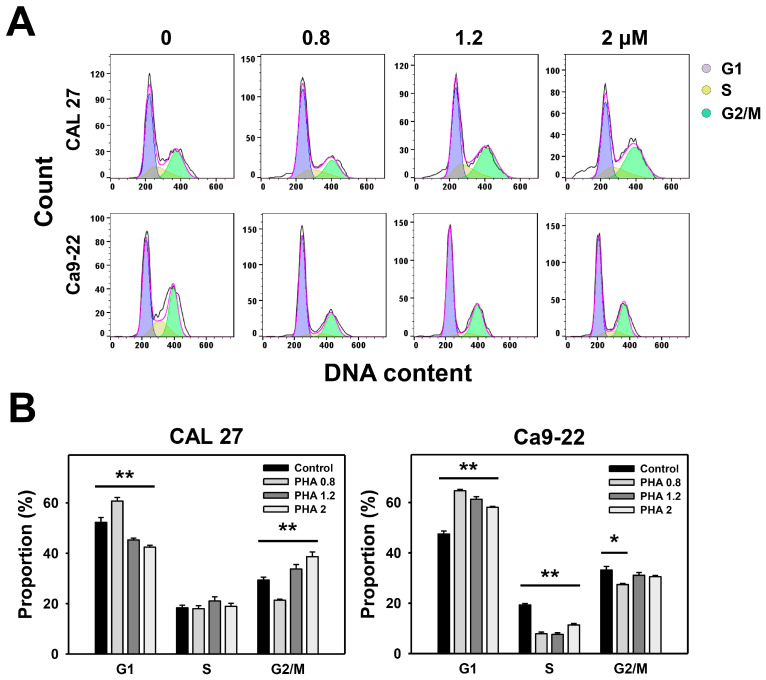
PHA affects the cell cycle progression of oral cancer cells. (**A**,**B**) Cell cycle distribution. Cells were exposed to PHA (0 (0.1% DMSO medium as control), 0.8, 1.2, and 2 μM) for 24 h, i.e., control, PHA 0.8, PHA 1.2, and PHA 2. Data, means ± SDs (*n* = 3 independent experiments); * and ** indicate *p* < 0.05 and 0.001.

The cell cycle was determined by the DNA content using flow cytometry. The flow cytometry histograms for cell cycle distribution of PHA-treated oral cancer cells were analyzed (Figure 2A). For CAL 27 cells, PHA only exhibited G1 accumulation at a low concentration (0.8 μM) and showed a decreased G1 accumulation at the other two higher concentrations (Figure 2B). Except for PHA 0.8 (PHA at 0.8 μM), other concentrations of PHA induced some G2/M arrest in CAL 27 cells (Figure 2B). For Ca9-22 cells, PHA mainly caused G1 arrest and decreased the S phase. PHA only decreased the G2/M phase at PHA 0.8. Therefore, PHA acting on different oral cancer cells may exhibit differential regulation of cell cycle progression.

### 2.3. PHA Enhances Annexin V-Monitored Apoptosis in Oral Cancer Cells

**Figure 3:**
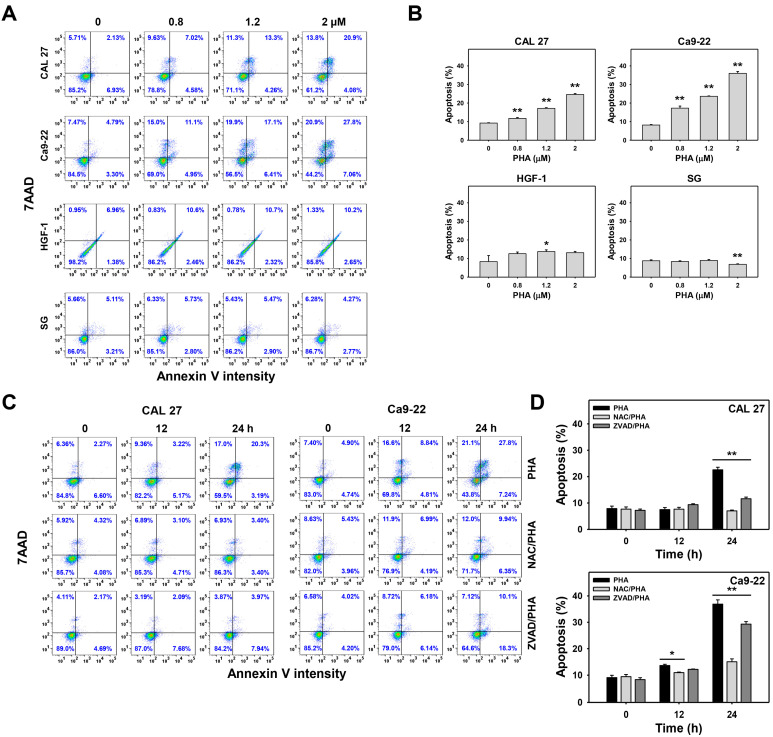
Annexin V intensity was increased after PHA treatment in oral cancer cells (CAL 27 and Ca9-22) but not for non-malignant oral cells (HGF-1 and SG). (**A**,**B**) Annexin V/7AAD analysis. Cells were exposed to 0 (0.1% DMSO medium as control), 0.8, 1.2, and 2 μM PHA for 24 h. 7AAD (+/−)/annexin V (+) (%) was counted for apoptosis (+) (%). (**C**,**D**) Suppression of PHA-induced apoptosis by NAC or ZVAD. To evaluate the recovery effect of NAC and ZVAD, NAC (10 mM for 1 h) or ZVAD (100 μM for 2 h) were added to cells before 12 and 24 h treatment with PHA (control (0.1% DMSO medium) and 2 μM), i.e., NAC/PHA or ZVAD/PHA. Data, means ± SDs (*n* = 3 independent experiments); * and ** indicate *p* < 0.05 and 0.001.

Annexin V can detect phosphatidylserine in the outer membrane, which is translocated from the inner membrane to the outer membrane for apoptotic cells. The apoptosis is proportional to annexin V intensity. The flow cytometry histograms for the annexin V/7–aminoactinmycin D (7AAD) detection of PHA-treated oral cancer cells were performed for apoptosis analysis (Figure 3A,C). PHA dose-responsively increased the annexin V (+) (%) population of oral cancer (CAL 27 and Ca9-22) cells (Figure 3B). In contrast, non-malignant oral cells (HGF-1 and SG) show little induction of annexin V (+) (%).

A 12 h time course of treatment for PHA 2 (PHA at 2 μM) did not change annexin V (+) (%) in CAL 27 cells, but it was moderately increased in Ca9-22 cells. A 24 h treatment for PHA 2 mainly increased the annexin V (+) (%) population of oral cancer cells (Figure 3D).

In addition, pretreatment with an oxidative stress inhibitor (NAC) substantially suppressed PHA-promoted annexin V (+) (%) populations for CAL 27 and Ca9-22 cells (Figure 3D). Similarly, pretreatment with an apoptosis inhibitor (ZVAD) moderately decreased PHA-promoted the annexin V (+) (%) population for oral cancer cells, indicating that PHA induced apoptosis in oral cancer cells. In comparison, NAC showed a higher suppression effect to PHA-induced apoptosis than ZVAD.

### 2.4. PHA Activates Apoptosis Signaling in Oral Cancer Cells

**Figure 4:**
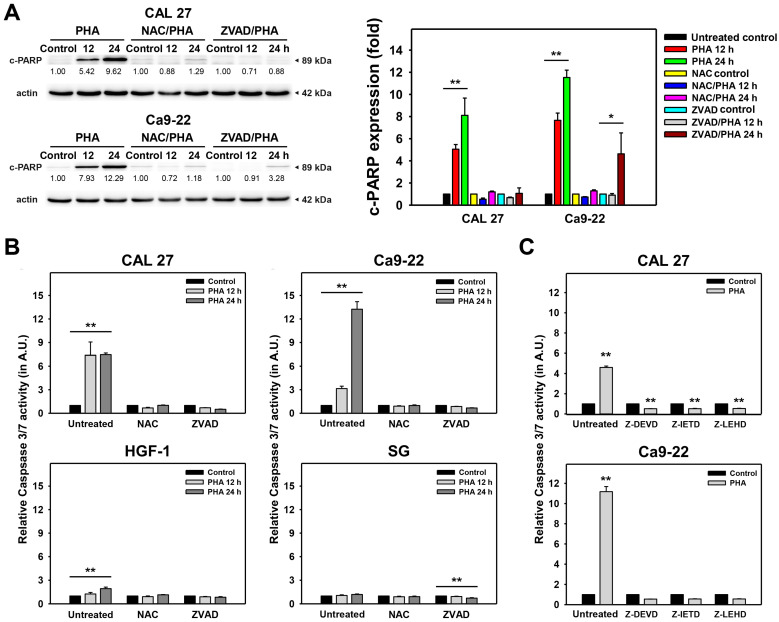
PHA enhances apoptotic protein expressions and activates Cas 3/7 in oral cancer cells and non-malignant oral cells. (**A**) Western blotting analysis. Cells were exposed to 0 (0.1% DMSO medium as PHA-untreated control) and 2 μM PHA for 12 and 24 h. c-PARP expression was detected for monitoring apoptosis. To evaluate the recovery effect of NAC and ZVAD, NAC (10 mM for 1 h) or ZVAD (100 μM for 2 h) were added to cells before 12 and 24 h treatment with PHA (control (0.1% DMSO medium) and 2 μM), i.e., NAC/PHA or ZVAD/PHA. (**B**) Suppression of PHA-induced Cas 3/7 activation by NAC or ZVAD. (**C**) Effects of Cas 3, 8, and 9 inhibitors on regulating Cas 3/7 activity. Either 10 μM for 2 h pretreatments with Z-DEVD (Cas 3 inhibitor), Z-IETD (Cas 8 inhibitor), or Z-LEHD (Cas 9 inhibitor), cells were exposed to 0 (0.1% DMSO medium as inhibitor-untreated control) and 2 μM of PHA for 24 h. Data, means ± SDs (*n* = 3 independent experiments); * and ** indicate *p* < 0.05 and 0.001.

The apoptosis detected by the annexin V/7AAD assay was further examined by western blotting and caspase 3/7 (Cas 3/7) assays. For western blotting, PHA increased the apoptotic cleaved poly (ADP-ribose) polymerase (c-PARP) expression in oral cancer cells (Figure 4A). This c-PARP induction was suppressed by NAC and ZVAD pretreatments, demonstrating that oxidative stress is involved in PHA-induced apoptosis in oral cancer cells.

Moreover, caspase signaling also plays a central role in regulating apoptosis. To examine the involvement of caspase signaling in PHA-induced apoptosis, a Cas 3/7 assay was performed and demonstrated that PHA induced Cas 3/7 activation (Figure 4B). Similar to c-PARP expression (Figure 4A), this Cas 3/7 activation was suppressed by NAC and ZVAD pretreatments.

To further examine the functions of Cas 3, 8, and 9 in regulating PHA-induced apoptosis, their specific inhibitors, such as Z-DEVD-FMK (Z-DEVD), Z-IETD-FMK (Z-IETD), and Z-LEHD-FMK (Z-LEHD), were applied for pretreatment (Figure 4C). After inhibitor pretreatment, PHA-induced apoptosis was suppressed by Cas 3, 8, and 9 inhibitors, demonstrating that intrinsic and extrinsic signaling is involved in the PHA-induced apoptosis of oral cancer cells.

### 2.5. PHA Enhances 2′,7′-Dichlorodihydrofluorescein Diacetate (H_2_DCFDA)-Monitored ROS Levels in Oral Cancer Cells

**Figure 5:**
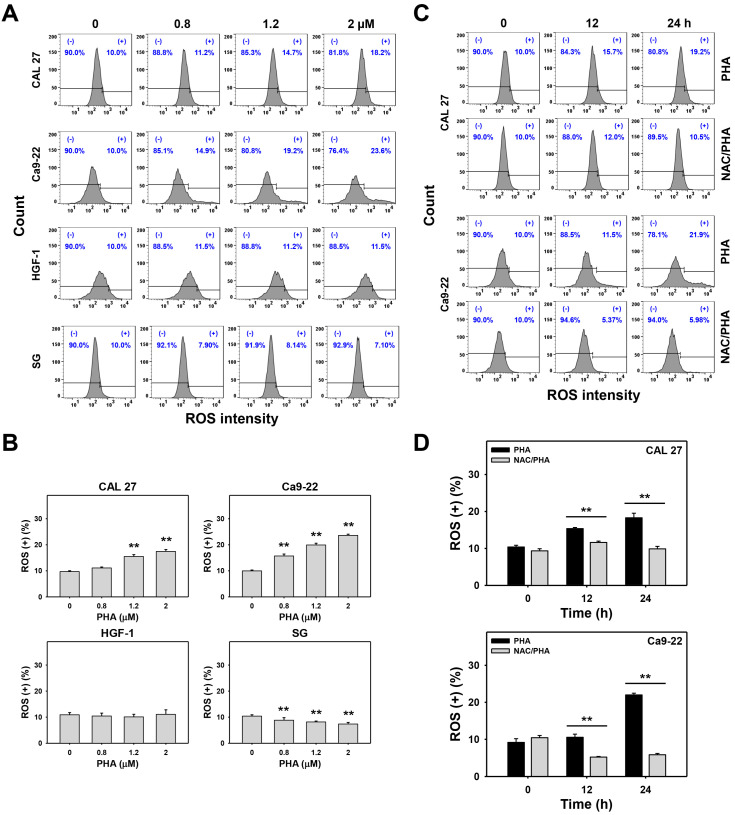
PHA enhances ROS intensity in oral cancer cells. (**A**,**B**) ROS analysis. Oral cancer (CAL 27 and Ca9-22) and non-malignant oral (HGF-1 and SG) cells were exposed to 0 (0.1% DMSO medium as control), 0.8, 1.2, and 2 μM PHA for 24 h. (+) population was counted for ROS (+) (%). (**C**,**D**) Suppression of PHA-induced ROS by NAC. To evaluate the recovery effect of NAC, NAC (10 mM for 1 h) was added to cells before 12 and 24 h treatment with PHA (control (0.1% DMSO medium) and 2 μM), i.e., NAC/PHA. Data, means ± SDs (*n* = 3 independent experiments); ** indicates *p* < 0.001.

The flow cytometry histograms for H_2_DCFDA detection of PHA-treated oral cancer cells were prepared for ROS analysis (Figure 5A,C). PHA increased the ROS (+) (%) population of oral cancer (CAL 27 and Ca9-22) cells in a dose-dependent manner (Figure 5B). In comparison, ROS induction for non-malignant oral cells (HGF-1 and SG) was minor.

In a time-course experiment, PHA increased the ROS (+) (%) population at 24 h exposure of oral cancer cells compared to the control and the 12 h treatment (Figure 5D). In addition, pretreatment with NAC substantially decreased the PHA-promoted ROS (+) (%) population in oral cancer cells (Figure 5D).

### 2.6. PHA Enhances MitoSOX^TM^ Red-Monitored Mitochondrial Superoxide (MitoSOX) Level in Oral Cancer Cells

**Figure 6:**
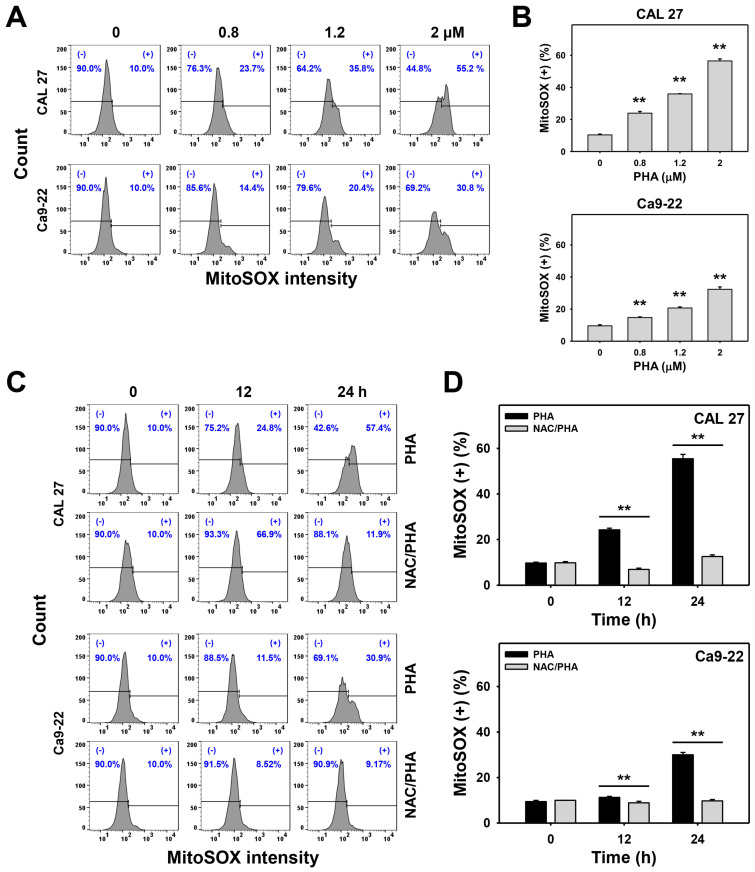
PHA enhances MitoSOX intensity in oral cancer cells. (**A**,**B**) MitoSOX analysis. Cells were exposed to 0 (0.1% DMSO medium as control), 0.8, 1.2, and 2 μM PHA for 24 h. The (+) population was counted for MitoSOX (+) (%). (**C**,**D**) Suppression of PHA-induced MitoSOX by NAC. To evaluate the recovery effect of NAC, NAC (10 mM for 1 h) was added to cells before 12 and 24 h treatment with PHA (control (0.1% DMSO medium) and 2 μM), i.e., NAC/PHA. Data, means ± SDs (*n* = 3 independent experiments); ** indicates *p* < 0.001.

After staining with the MitoSOX-detecting dye (MitoSOX^TM^ Red), the flow cytometry histograms for MitoSOX analysis of PHA-treated oral cancer cells were performed (Figure 6A,C). PHA increased the MitoSOX (+) (%) population of oral cancer (CAL 27 and Ca9-22) cells in a dose-dependent manner (Figure 6B), suggesting that PHA induced MitoSOX generation in oral cancer cells. PHA promoted a higher MitoSOX (+) (%) population at 24 h exposure to oral cancer cells than in the control and 12 h treatment (Figure 6D). In addition, pretreatment with NAC substantially suppressed the PHA-promoted MitoSOX (+) (%) population in oral cancer cells (Figure 6D).

### 2.7. PHA Decreases DiOC_2_(3)-Monitored Mitochondrial Membrane Potential (MitoMP) in Oral Cancer Cells

**Figure 7:**
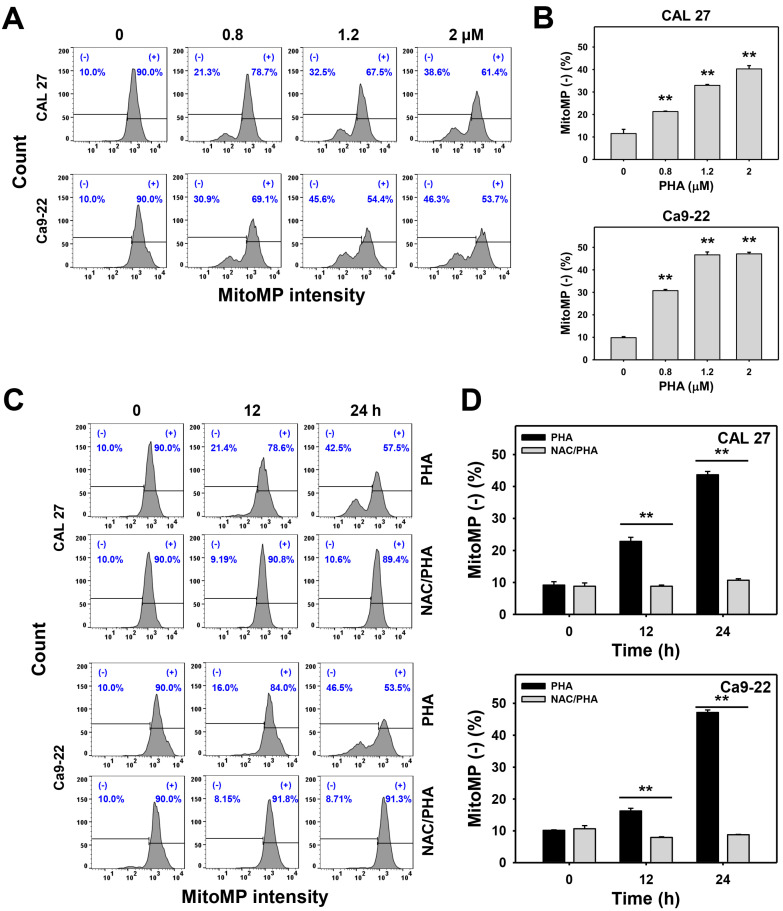
PHA enhances the MitoMP intensity in oral cancer cells. (**A**,**B**) MitoMP analysis. Cells were exposed to 0 (0.1% DMSO medium as a control), 0.8, 1.2, and 2 μM PHA for 24 h. (−) population was counted for MitoMP (−) (%). (**C**,**D**) Suppression of PHA-induced MitoMP depletion by NAC. To evaluate the recovery effect of NAC, NAC (10 mM for 1 h) was added to cells before 24 h treatment with PHA (control (0.1% DMSO medium) and 2 μM), i.e., NAC/PHA. Data, means ± SDs (*n* = 3 independent experiments); ** indicates *p* < 0.001. The positive control for MitoMP depletion is provided in the Supplementary Figure S1.

Staining with the MitoMP-detecting dye DiOC_2_(3), the flow cytometry histograms for MitoMP analysis of PHA-treated oral cancer cells were performed (Figure 7A,C). The increment of the MitoMP (−) (%) population indicated that MitoMP was decreased after drug treatment. PHA increased the MitoMP (−) (%) population of oral cancer (CAL 27 and Ca9-22) cells in a dose- and time-responsive behavior (Figure 7B,D). In addition, pretreatment with NAC substantially suppressed the PHA-promoted MitoMP (−) (%) population for oral cancer cells (Figure 7D).

### 2.8. PHA Enhances Antibody-Monitored γH2AX Levels in Oral Cancer Cells

**Figure 8:**
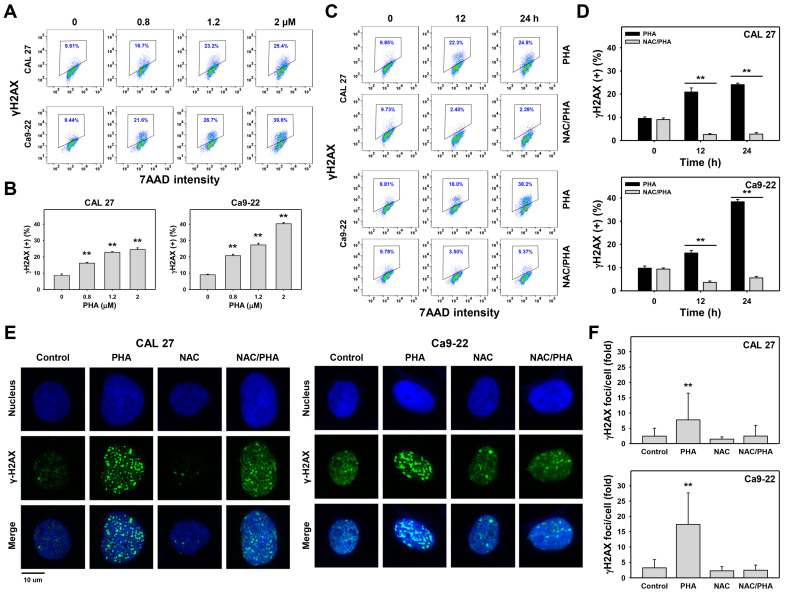
PHA promotes γH2AX intensity and foci number in oral cancer cells. (**A**,**B**) γH2AX analysis. Cells were exposed to 0 (0.1% DMSO medium as control), 0.8, 1.2, and 2 μM PHA for 24 h. (+) population was counted for γH2AX (+) (%). (**C**,**D**) Suppression of PHA-induced γH2AX by NAC. To evaluate the recovery effect of NAC, NAC (10 mM for 1 h) was added to cells before 12 and 24 h treatment with PHA (control (0.1% DMSO medium) and 2 μM), i.e., NAC/PHA. Data, means ± SDs (*n* = 3 independent experiments). (**E**,**F**) γ-H2AX foci analysis. Cells were immunostained by γH2AX primary antibody/Alexa Fluor 488-conjugated secondary antibody and counterstained with Hoechst 33342. γH2AX foci were counted as foci per cell. Slides were photographed at 400× magnification. Data, means ± SDs (*n* = 30 cells); ** indicates *p* < 0.001.

The flow cytometry histograms for antibody detection of PHA-treated oral cancer cells were performed for γH2AX analysis (Figure 8A,C). PHA increased the γH2AX (+) (%) population of oral cancer (CAL 27 and Ca9-22) cells in a dose- and time-responsive manner (Figure 8B,D). In addition, pretreatment with NAC substantially suppressed the PHA-promoted γH2AX (%) population for oral cancer cells (Figure 8D).

Moreover, γH2AX flow cytometry expression was further validated by γH2AX foci immunofluorescence (Figure 8E). The population for γH2AX foci was higher in PHA-treated oral cancer cells than in the control. In addition, pretreatment with NAC substantially suppressed PHA-promoted γH2AX foci (Figure 8F).

### 2.9. PHA Inhibits mRNA Expressions of DNA Repair Genes in Oral Cancer Cells

**Figure 9:**
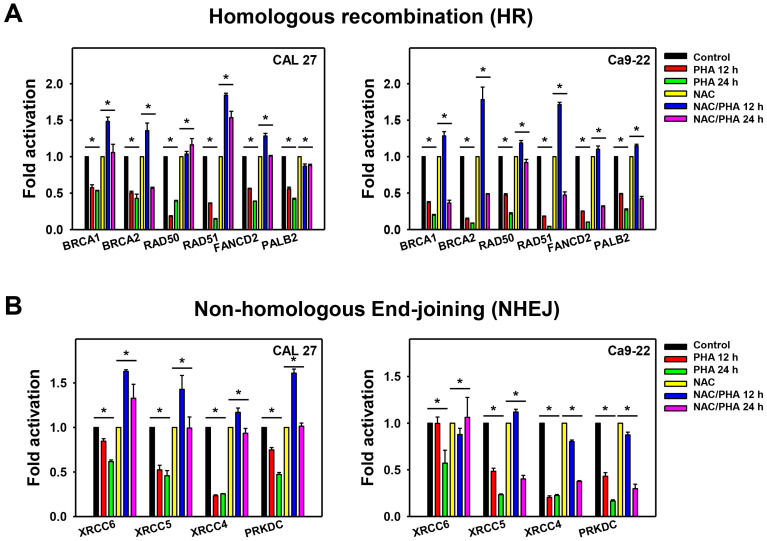
PHA inhibits mRNA expressions of DNA repair genes in oral cancer cells. NAC (10 mM for 1 h) was added to cells before 0, 12, and 24 h treatment with PHA (control (0.1% DMSO medium) and 2 μM), i.e., control (PHA-untreated and NAC only), PHA 12 h vs. NAC/PHA 12 h, PHA 24 h vs. NAC/PHA 24 h. (**A**,**B**) mRNA expression of HR and NHEJ genes. mRNA expressions were adjusted with the *GAPDH* gene. Data, means ± SDs (*n* = 3 independent experiments); * indicates *p* < 0.05.

Inhibitions of the DNA repair machinery are prone to accumulate DNA damage [23]. Hence, DNA repair signaling was examined. The mRNA expressions for DNA repair signaling [24], including HR-associated genes (BRCA1 DNA repair-associated (*BRCA1*), *BRCA2*, RAD50 double-strand break repair protein (*RAD50*), RAD51 recombinase (*RAD51*), FA complementation group D2 (*FANCD2*), and partner and localizer of BRCA2 (*PALB2*)) and NHEJ-associated genes (X-ray repair cross-complementing 6 (*XRCC6*), *XRCC5*, *XRCC4*, and protein kinase, DNA-activated, catalytic subunit (*PRKDC*)), were examined for PHA-incubated oral cancer cells. After 12 and 24 h PHA incubation, the fold activation of mRNA expressions for these DNA repair genes (HR and NHEJ) in oral cancer (CAL 27 and Ca9-22) cells were decreased as compared to the control (Figure 9A,B). In addition, pretreatment with NAC recovered PHA-suppressed mRNA (Figure 9A,B) expressions for DNA repair genes (HR and NHEJ) of the oral cancer cells.

## 3. Discussion

Reports of the anticancer effects and drug safety of *P. peruviana*-derived PHA are rare. To date, only two reports have been published that have mentioned the use of PHA in cancer [8,16]. The present study demonstrated that PHA exhibits selective antiproliferation, oxidative stress, and apoptosis in oral cancer cells when compared to non-malignant oral cells. Although PHA-induced γH2AX flow cytometry-detected DNA damage was reported in breast cancer cells before [16], the suppressing effects of the DNA repair of PHA are reported here for the first time.

### 3.1. PHA Exhibits Selective ROS Generation and Selective Oxidative Stress-Dependent Anitproliferation in Oral Cancer Cells

Drug-induced oxidative stress has the potential antiproliferation of cancer cells [25,26,27]. However, the oxidative stress changes in normal cells were sometimes not investigated. In the present study, PHA generated more ROS in two oral cancer cell lines (CAL 27 and Ca9-22) than in two non-malignant oral cell lines (HGF-1 and SG) (Figure 5), suggesting that PHA exhibits a selective ROS generation ability to oral cancer cells.

ROS exhibits a dual role in regulating physiological and pathological function [28,29]. ROS improves tumor invasion, metastasis, and angiogenesis in cancer cells [28,30]. In contrast, immense ROS accumulation suppresses tumor growth [28]. This PHA-induced selective ROS generation is expected to cause massive ROS accumulation and induce selective antiproliferation in oral cancer cells but not for non-malignant oral cells (Figure 1). In addition, the ROS scavenger NAC can revert the ROS induction and antiproliferation of oral cancer cells following PHA treatment. These results suggest that PHA-induced selective antiproliferation to oral cancer cells depends on oxidative stress. Its non-cytotoxicity to non-malignant oral cells also supports the drug safety of PHA.

The anticancer effects of PHA were reported in prostate and renal cancer cells [8] by providing IC_50_ values without investigating anticancer mechanisms. The IC_50_ concentrations for 72 h PHA in the MTS assay for prostate (LNCaP) and renal (ACHN) cancer cells were 0.11 and 1.0 μM, respectively. This study also found that PHA showed low cytotoxicity (>2 μM) to normal cells. The latter held for non-tissue matched human foreskin fibroblasts (HEF), which were applied as a control [8].

The IC_50_ concentrations for PHA at 24 h in an ATP assay for breast cancer cells (SKBR3, MCF7, and MDA-MB-23) were 4.18, 3.12, and 6.15 μM [16], respectively. In the present study, IC_50_ concentrations for PHA at 24 h in an ATP assay for the oral cancer cells (CAL 27 and Ca9-22) were 0.86 and 1.61 μM, respectively. Accordingly, PHA is more sensitive to oral cancer cells than breast cancer cells; however, the normal cell response was not examined in the breast cancer study [16]. When compared to the clinical anticancer drug cisplatin, IC_50_ concentrations for cisplatin at 24 h in ATP assay for the oral cancer cells (CAL 27 and Ca9-22) were 6.02 and 11.08 μM (Figure 1D), respectively. Therefore, PHA is more effective than cisplatin in the antiproliferation of oral cancer cells.

### 3.2. PHA Exhibits Oxidative Stress-Dependent Selective Apoptosis in Oral Cancer Cells

In addition to ROS generation, PHA also evoked other oxidative stresses such as MitoSOX generation and MitoMP depletion in oral cancer cells (Figure 6 and Figure 7). PHA also induced oxidative stress and apoptosis in a breast cancer study [14]. For PHA-treated breast cancer cells, both extrinsic and intrinsic apoptosis signaling such as Cas 8 and Cas 9 and the apoptosis executor Cas 3 were activated by PHA in western blotting analysis without further validation by their specific inhibitors [16].

Similarly, PHA induced apoptosis (Figure 3) and triggered apoptosis signaling for c-PARP in western blotting and Cas 3/7 activity assays (Figure 4). In contrast, PHA did not activate Cas 3/7 activity in non-malignant oral cells, suggesting that PHA induces selective apoptosis in oral cancer cells rather than in non-malignant oral cells. In addition, the pancaspase inhibitor (ZVAD) supported that PHA-induced apoptosis contributed to PHA-induced antiproliferation in oral cancer cells (Figure 4B). Moreover, these extrinsic, intrinsic, and executor proteins for apoptosis signaling in oral cancer cells were further confirmed by Cas 3/7 assays using the specific Cas 8, Cas 9, and Cas 3 inhibitors (Figure 4C). Finally, both PHA-induced oxidative stress and apoptosis were suppressed by NAC. Accordingly, PHA causes selective apoptosis in an oxidative stress-dependent manner in oral cancer cells.

### 3.3. PHA Induces Oxidative Stress-Dependent DNA Damage and Inhibits Oxidative Stress-Dependent DNA Repair in Oral Cancer Cells

PHA-induced γH2AX phosphorylation was reported in breast cancer cells by flow cytometry [16], which was similar to oral cancer cells in the present study (Figure 8A–D). However, the flow cytometry-detected γH2AX phosphorylation was not specific to the DNA damage. In addition to target DNA double-strand break (DSB) sites, some free γH2AX may have existed in the cytoplasm. Both of them were detected by flow cytometry.

To exclude the non-specific detection, we further detected the γH2AX foci in PHA-treated oral cancer cells by immunofluorescence. The γH2AX foci number was increased after PHA treatment. Both flow cytometry-detected γH2AX and γH2AX foci were suppressed by NAC. These results suggest that PHA induces oxidative stress-dependent DNA damage in oral cancer cells. However, the DNA damage evidence of PHA was still weak in the present study. It warrants a detailed assessment of DNA damage by other assays such as the alkaline comet assay in the future.

When the DNA repair is dysfunctional, the anticancer effects of drugs on cancer cells may be improved [18,31,32]. For example, prodigiosin caused antiproliferation by suppressing RAD51-mediated HR repair in breast cancer cells [31]. Although the DNA damage effects of PHA have been reported in breast cancer cells based on γH2AX flow cytometry [16], its action on DNA repair has not been investigated as yet.

In the present study, PHA suppressed mRNA expressions for HR and NHEJ repair systems (Figure 9). This repair inhibition ability was suppressed by NAC. These results suggest that PHA induces oxidative stress-dependent suppression of DNA repair in oral cancer cells. This DNA repair ability may help overcome drug resistance [18]. Notably, this study cannot exclude the possibility that other DNA repair pathways may be involved in PHA treatment acting on oral cancer cells. Moreover, the DNA repair-suppressing ability of PHA was demonstrated by mRNA expressions. In a future study, the protein expressions of DNA repair genes after PHA treatment should be detected.

### 3.4. Potential Targets of PHA

We found that PHA inhibited the DNA repair process by blocking mRNA expressions of DNA repair genes, but the interaction between PHA and DNA repair enzymes is unclear. A molecular docking study for several withanolide analogs identified heat shock protein 90 (HSP90) as a potential target [33]. HSP90 can regulate DNA repair proteins [34] and apoptosis [35]. However, the role of HSP90 in *P. peruviana*-derived withanolide (PHA) remains unclear. Notably, p53 and phosphatidylinositol-4,5-bisphosphate 3-kinase catalytic subunit alpha (PI3KCA) are commonly mutated in oral cancer [36]. Both p53 [37] and PI3KCA [38] can regulate DNA damage response or repair. For example, p53 modulates HR and NHEJ repair signaling [39]. PI3KCA can interact with NHEJ proteins and leads to apoptosis [40]. Hence, the impacts of p53 and PI3KCA in the selective killing effects of PHA need further examination. Moreover, HSP90 can regulate p53 activity [41], and PI3K [42] is a HSP90 client. Therefore, the role of HSP90 and its downstream clients warrant a detailed investigation of the PHA mechanism of action in the antiproliferation of oral cancer cells.

## 4. Materials and Methods

### 4.1. PHA Preparation

PHA was isolated from *Physalis peruviana* roots, and its chemical profile was confirmed as described in our previous work [16]. The purity of PHA was examined by analytical HPLC (>99%) before the experiments.

### 4.2. Cell Cultures and Inhibitors for Oxidative Stress and Apoptosis

Oral cancer (CAL 27 and Ca9-22) and non-malignant (HGF-1) oral cell lines were collected from the public cell banks JCRB and ATCC. Another non-malignant oral cell line was included, the human normal gingival epithelial Smulow–Glickman (SG) cell line. SG cells are well characterized [43,44] and applied to examine the cytotoxicity of several dental materials [45,46] and to evaluate the safety of anti-oral cancer drugs [47,48]. All cell lines were cultured at 5% CO_2_ and humidified in 37 °C atmospheres. Cells were maintained by mixtures of Dulbecco’s Modified Eagle Medium (DMEM) and F12 (Gibco, Grand Island, NY, USA) at 3:2 (oral cancer cells) and 4:1 (HGF-1 and SG cells) [49], supplemented with 10% fetal bovine serum and routine culture antibiotics.

The oxidative stress inhibitor NAC (Sigma-Aldrich, St. Louis, MO, USA) was chosen [50,51,52,53]. Inhibitors for general caspases (pancaspase), and the specific caspases 3, 8, and 9, such as ZVAD, Z-DEVD, Z-IETD, and Z-LEHD (Selleckchem.com; Houston, TX, USA), were used.

### 4.3. ATP-Based Cell Viability

After seeding overnight, drug treatments were performed as indicated in the figure legend. Subsequently, cell survival was examined by the ATPlite luminescence product (PerkinElmer Life Sciences, Boston, MA, USA) [49]. According to the user manual, cell lysate was incubated with the substrate in darkness for 5 min and read by a microplate luminometer (Berthold Technologies GmbH & Co., Bad Wildbad, Germany).

### 4.4. Cell Cycle

After seeding overnight, drug treatments were performed as indicated in the figure legend. Subsequently, cells were fixed and washed before being stained with 7AAD (Biotium; Hayward, CA, USA), i.e., 5 μg/mL (37 °C, 30 min) [54]. Subsequently, the intensity of 7AAD was determined by a Guava easyCyte flow cytometer (Luminex, Austin, TX, USA) under the red channel. The cell cycle phase was calculated by FlowJo software (Becton-Dickinson, Franklin Lakes, NJ, USA).

### 4.5. Apoptosis

After seeding overnight, drug treatments were performed as indicated in the figure legend. Subsequently, apoptosis of non-fixed cells was determined by annexin V/7AAD, Caspase-Glo^®^ 3/7, and western blotting as follows. Annexin V-FITC (1:1000)/7AAD (1 μg/mL) kit (Strong Biotech Corporation, Taipei, Taiwan) was added to cell suspensions at 37 °C for 30 min and applied to flow cytometry analysis under the green/red channels [49]. The positive (+) population was calculated by FlowJo software and regarded as the (+) intensity for apoptosis level, as indicated in the figure legend.

The apoptosis-executing enzyme Cas 3/7 was activated by apoptosis. The activity of Cas 3/7 was determined by Caspase-Glo^®^ 3/7 kit (Promega; Madison, WI, USA) [55]. The Cas 3/7 tetrapeptide substrate (DEVD) can react with active Cas 3/7. After cutting by active Cas 3/7, DEVD became a luminogen, and a microplate luminometer measured its intensity.

Apoptosis signaling protein expressions were detected by western blotting. Apoptosis antibodies included c-PARP (Cell signaling #5625; Danvers, MA, USA). β-actin (Sigma-Aldrich; St. Louis, MO, USA) was used to detect the loading control [10]. The remaining information was mentioned previously [49].

### 4.6. ROS

After seeding overnight, drug treatments were performed as indicated in the figure legend. Subsequently, non-fixed cells were processed with the nonfluorescent H_2_DCFDA staining (Sigma-Aldrich) at 10 μM (37 °C, 30 min) in darkness [49]. The ROS-activated dye became a fluorophore and was detected by the Guava easyCyte flow cytometer under the green channel. Its intensity was calculated by FlowJo software. The positive (+) population was calculated by FlowJo software and regarded as the (+) intensity for ROS level, as indicated in the figure legend.

### 4.7. MitoSOX

After seeding overnight, drug treatments were performed as indicated in the figure legend. Subsequently, non-fixed cells were processed with MitoSOX Red (Invitrogen, Eugene, OR, USA) at 50 nM (37 °C, 30 min) in darkness [49]. The MitoSOX activated dye became a red fluorophore and was detected by the Guava easyCyte flow cytometer under the red channel. The positive (+) population was calculated by FlowJo software and regarded as the (+) intensity for the MitoSOX level, as indicated in the figure legend.

### 4.8. MitoMP

After seeding overnight, drug treatments were performed as indicated in the figure legend. Subsequently, non-fixed cells were processed with DiOC_2_(3) (Invitrogen) at 5 nM (37 °C, 20 min) in darkness [56]. The MitoMP activated dye became a green fluorophore and was detected by the Guava easyCyte flow cytometer under the green channel. The negative (−) population was calculated by FlowJo software and regarded as the (−) intensity for MitoMP decreasing level as indicated in the figure legend.

### 4.9. DNA Damage

After seeding overnight, drug treatments were performed as indicated in the figure legend. Subsequently, DNA damages were determined by monitoring the expression of γH2AX using flow cytometry and immunofluorescence as following.

For γH2AX level monitoring by flow cytometry [49], cells were processed with fixation and antibody incubation (γH2AX primary antibody 1:500 (Santa Cruz, Biotechnology, Santa Cruz, CA, USA) and Alexa Fluor 488 secondary antibody 1:10,000 (Cell Signaling Technology). Following 7AAD (5 μg/mL) staining, the stained cells were used for flow cytometry under the green/red channels. The positive (+) population was calculated by FlowJo software and regarded as the (+) intensity for the γH2AX level, as indicated in the figure legend. This is a fast screening for γH2AX level, but it may detect some non-specific DNA damage.

Alternatively, γH2AX foci being proportional to the number of DNA damage sites provide a more direct assessment of DNA damage. To exclude the non-specific detection in flow cytometry, γH2AX foci in PHA-treated oral cancer cells were assessed by immunofluorescence. For γH2AX foci monitoring, all steps were performed at room temperature. In general, cells were fixed with 4% paraformaldehyde for 10 min and washed with PBS. After permeabilization with 0.1% PBS Triton X-100 for 5 min, cells were washed with PBS and then blocked with 1% BSA in PBS for 1 h. After blocking, cells were incubated with γH2AX antibody (Santa Cruz, Biotechnology, CA, USA) (1:400) for 1 h. After primary antibody incubation, cells were washed with PBS and incubated with Alexa Fluor 488-conjugated secondary antibody (1:500 dilution) and bisBenzimide H33342 trihydrochloride (Sigma-Aldrich) (1:1000 dilution) for 1 h. Cells were washed three times with PBS and mounted with Dako Fluorescence Mounting Medium (Invitrogen, Grand Island, NY, USA). Slides were photographed using a DMi8 inverted microscope (Leica Microsystems, Wetzlar, Germany). γH2AX foci were analyzed by the open software Image J.

### 4.10. mRNA Expressions for DNA Repair Genes (HR and NHEJ)

After seeding overnight, drug treatments were performed as indicated in the figure legend. Subsequently, RNA and cDNA were prepared for quantitative reverse transcription-PCR (qRT-PCR) by performing a touch-down program as described [57]. Both HR and NHEJ DNA repair genes were tested (Table 1). HR gene expressions [24] were analyzed, including *BRCA1*, *BRCA2*, *RAD50*, *RAD51*, *FANCD2*, and *PALB2*. NHEJ gene expressions [24] were analyzed, including *XRCC6*, *XRCC5*, *XRCC4*, and *PRKDC*. mRNA expression was analyzed by the 2^−ΔΔCt^ method [58] compared to the *GAPDH* gene [59,60].

### 4.11. Statistical Analysis

Except for the Western blotting results, which were analyzed by the Student’s *t*-test, the significance for multi-comparisons in the other data was analyzed by one-way ANOVA and the Tukey’s HSD post hoc test (JMP software, SAS Institute Inc., Cary, NC, USA). All results were analyzed from 3 independent experiments; * and ** indicate *p* < 0.05 and 0.001.

## 5. Conclusions

The anticancer effects of *P. peruviana*-derived physapruin A (PHA) were rarely reported, especially not for selective antiproliferation in oral cancer cells. The present study confirmed that PHA effectively suppressed oral cancer cell proliferation and showed good safety to non-malignant oral cells, indicating that PHA exhibits selective antiproliferation effects on oral cancer cells. Mechanistically, PHA induced more ROS and apoptosis in oral cancer cells than in non-malignant oral cells, indicating that PHA has selective ROS and apoptosis-inducing effects on oral cancer cells. PHA also induced several oxidative stress indicators in oral cancer cells, such as MitoSOX generation and MitoMP depletion. In addition to PHA-induced double-strand break DNA damage, its DNA repair system including HR and NHEJ was inhibited by PHA. Such PHA-associated modulations were suppressed by NAC, indicating the oxidative stress dependence on PHA function in oral cancer cells. Therefore, PHA is a novel selective antiproliferation agent with low toxic side effects on non-malignant oral cells. It shows potential for multiple effects, including increasing oxidative stress, apoptosis, DNA damage, and impairing DNA repair in oral cancer cells.

## Figures and Tables

**Table 1:** Basic information for DNA repair by HR and NHEJ genes.

Genes	Forward Primers (5′ → 3′)	Reverse Primers (5′ → 3′)	Accession No.
*BRCA1*	GAACCAGGAGTGGAAAGGTCAT	CAGGTAAGGGGTTCCCTCTAGA	NM_007294.4
*BRCA2*	GAGCATACCCTATACAGTGGATGG	CTCTTCACTGAAATAACCCTCAAGG	NM_000059.4
*RAD50*	AAAACAGCGAGCCATGCTG	TATGCTTTGCCTCATGGGC	NM_005732.4
*RAD51*	TGGCAGTGGCTGAGAGGTATG	CCACTGCTACACCAAACTCATCAG	NM_002875.5
*FANCD2*	GGATGAGGAAGCCAGTATGGG	CTTGGTGGTGAGGTCCTTGC	NM_033084.6
*PALB2*	CGCAGAGGTTCCAGTATTACAGATAGT	GCCTCCTCCATCTTCTGCAAAC	NM_024675.4
*XRCC6*	AGTCGCTGGTGATTGGGAGC	AGACCAGCTGGAAGCCTGGA	NM_001469.5
*XRCC5*	CAGCTTTGAGGAAGCGAGTAACC	TGGGGGCCAGAAACTTTTTG	NM_021141.4
*XRCC4*	CATGGACTGGGACAGTTTCTGA	GGAACCAAGTCTGAATGAGACATC	NM_003401.5
*PRKDC*	CAGTGGTCCTTCCAAAGGGC	CATTCTCTTGTTCCCCAACAGTCT	NM_006904.7
*GAPDH*	CCTCAACTACATGGTTTACATGTTCC	CAAATGAGCCCCAGCCTTCT	NM_002046.7

## Data Availability

Data are contained within the article.

## Supplementary Materials

The following are available online at https://www.mdpi.com/article/10.3390/ijms23168839/s1.
