# Supplementary material for: Physapruin A Enhances DNA Damage and Inhibits DNA Repair to Suppress Oral Cancer Cell Proliferation
Source: Int J Mol Sci. 2022 Aug 9;23(16):8839. doi: 10.3390/ijms23168839 (PMC9408722; doi:10.3390/ijms23168839)
Supplement: Supplementary file 1 [file ijms-23-08839-s001.zip › ijms-1834006-supplementary.pdf]

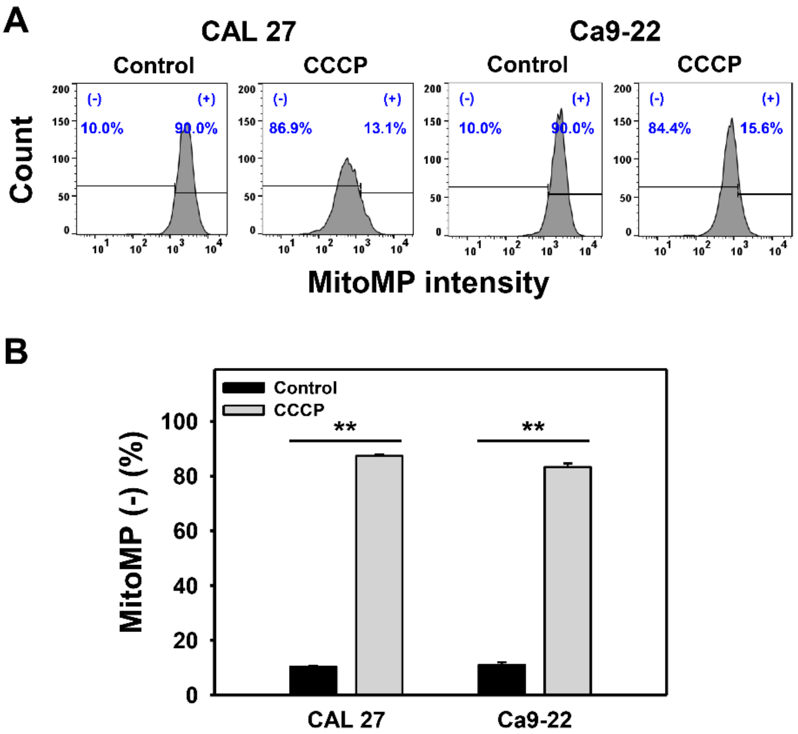

Supplementary Figure S1. Positive control for MitoMP depletion in oral cancer cells. (**A** and **B**) MitoMP analysis. Cells were exposed to 0 (control) and 50 μM carbonyl cyanide m-chlorophenyl hydrazone (CCCP) for 20 min. (-) population was counted for MitoMP (-) (%). Data, means ± SDs (n = 3). ** indicated $p < 0.001$.
